# Role of platelet indices, glycemic control and hs-CRP in pathogenesis of vascular complications in type-2 diabetic patients

**DOI:** 10.12669/pjms.291.2592

**Published:** 2013

**Authors:** Farah Jabeen, Asher Fawwad, Husan Afroz Rizvi, Faraz Alvi

**Affiliations:** 1 Farah Jabeen, M.Sc, MS. Assistant Professor, Department of Biochemistry, Jinnah University for Women, V-C Nazimabad, Karachi, Pakistan.; 2 Dr. Asher Fawwad, MBBS, M.Phil, Assistant Professor, Research Department, Baqai Institute of Diabetology and Endocrinology, Baqai Medical University, Karachi, Pakistan.; 3 Husan Afroz Rizvi, M.Sc, PhD (Germany), Professor, Department of Biochemistry, Jinnah University for Women, V-C Nazimabad, Karachi, Pakistan.; 4 S. Faraz Danish Alvi, MBBS, Research Officer, Research Department, Baqai Institute of Diabetology and Endocrinology, Baqai Medical University, Karachi, Pakistan.

**Keywords:** Diabetes Mellitus Type-II, hs-CRP, Micro / Macro vascular complications

## Abstract

***Objectives:*** Alteration in platelet morphology and functions are associated with pathological processes and increased risk of vascular complications in patients with diabetes. The purpose of the study was to find the correlation between platelet indices with fasting blood glucose, HbA1c and hs-CRP level in pathogenesis of vascular complications in type 2 diabetic patients.

***Methodology:*** The study has been carried out on 51 Type 2 Diabetics and 55 age and sex matched healthy control subjects. Fasting blood glucose (FBG), Glycosylated hemoglobin (HbA_1c_), high sensitivity C- reactive protein (hs-CRP) level and platelet indices including Platelet count (PLT), Plateletcrit (PCT), Mean platelet volume (MPV), Platelet distribution width (PDW) were estimated and compared with normal subjects. The results were evaluated statistically.

***Results:*** The study demonstrated that FBG, HbA1c, MPV, PDW and hs-CRP were statistically higher in diabetics in comparison with control subjects (P is less than 0.05). Positive correlation of FBG with HbA1c (r is equal to 0.993, P is equal to 0.0001), PLT with PCT (r is equal to 0.922, P is equal to 0.0001) and MPV with PDW (r is equal to 0.332, P is equal to 0.024) was found in diabetics.

***Conclusion:*** The poor glycemic control is positively correlated with high HbA1c level. The increased values of MPV, PDW and elevated hs-CRP level may also serve as confirmatory test in finding risk of developing complications.

## Introduction

 Diabetes mellitus, is characterized by varying degree of hyperglycemia accompanied with the biochemical alterations in carbohydrate, protein and lipid metabolism. Generally the injurious effects of hyperglycemia are categorized as macro vascular complications (coronary artery disease, peripheral arterial disease, and stroke) and micro vascular complications (diabetic nephropathy, neuropathy, and retinopathy).^1^ Altered platelet morphology and functions have been reported in diabetic patients and are linked with the pathological processes and high risk of vascular disease.^2^

 Platelets play a key role in the genesis of thrombosis, majority of the studies that had been conducted in order to correlate coagulation abnormalities were linked with platelet functions. The significant increase in platelet count and MPV correlated with increase adhesiveness, aggregation and greater exposure of glycoprotein receptor on platelet membrane and increase binding of fibrinogen, which could alter platelet metabolism and inter platelet signaling pathway that eventually leads to impairment of various metabolic pathways like increase calcium metabolism, ADP production and thromboxane (TXA_2_) synthesis and release.^3^ Platelet indices (PLT, PCT, MPV, PDW) are determinant of platelet functionality, among which increased mean platelet volume (MPV) and platelet distribution width (PDW) were found the important contributory factors causing thromboembolic complications. Mean platelet volume (MPV) is a determinant of platelet functionality and increased MPV is related with high risk of cardiovascular disease like myocardial infarction, stroke and transient ischemic attacks.^4^ Increased platelet sensitivity have direct consequence in diabetes, might be related with release of contents from platelet granules which in turn may lead to the making of a platelet volume gradient, increased platelet turnover rate and reduction in survival of platelets in diabetic individuals.

 Several studies have revealed that platelet with increased number and size possibly affecting the platelet distribution width contributing in pathogenesis of vascular complications. Hyperactivity of platelets have important role in initiation of atherosclerotic lesions and coronary thrombogenesis. Larger platelets are more active enzymatically and metabolically and have higher thrombotic ability as compared to small sized platelets.^5^ Thrombogenesity of large platelets may put the patient at a higher risk. Identification of such Patients with larger platelets can easily be made during routine hematological analysis which is simple, easy and cost effective tool that could possibly benefit for preventive actions. A large number of patients with type 2 DM suffer from preventable macro vascular complications. The prevalence of diabetic micro vascular complications is greater in people with poor control of glucose level, long term duration of diabetes, an elevated blood pressure and obesity.^6^

 During the last few years, the role of hs-CRP in inflammatory states has been reported. The hs-CRP is a protein of an acute phase secreted by the liver as well as by other tissues in response to any inflammatory condition. Furthermore, anciently the hs- CRP was considered as an inert biomarker of inflammation now modified by evidences of its direct role in pro-inflammatory activity. Now CRP is considered one of the most important pro-atherosclerotic mediators.^7^ There is a need to develop risk factor modification interventions to reduce the impact of complications in diabetic patients.

## Methodology

 The study was conducted on 51 type 2 diabetic patients (28 males & 23 females) between the age group 35-65 years who were registered at Baqai Institute of Diabetology and Endocrinology, Karachi, Pakistan. Fifty five (22 males and 33 females) age and sex matched control subjects were also selected from the general population at random for comparison. Ethical approval was taken from the institutional review board (IRB) prior to the commencement of the study. Informed consent was taken from each individual at the time of recruitment in the study. The patients were diagnosed type 2 diabetes using the ADA criteria i.e. fasting blood glucose (FBG) equal or greater to 126 mg/dl were included in the study. Each patient was interviewed at the time of taking blood sample and all necessary information required for the study was recorded. Moreover, the patient’s hematological and clinical reports of cardiac, renal and liver function tests were also observed. The patients who had any recent clinical evidence of cardiac, renal or liver dysfunctions and any hemoglobinopathy were excluded from the study.

 Blood samples were collected in tubes with EDTA as anticoagulant and analyzed within two hours of venepuncture for platelet indices and HbA_1c_. Plasma was also separated and analyzed for other biochemical parameters such as fasting blood glucose and CRP levels. Platelet indices such as platelet count (PLT), Plateletcrit (PCT), mean platelet volume (MPV) and platelet distribution width (PDW) were analyzed by using hematological analyzer ABX micros 60 fully automated analyzer HORIBA (France). Fasting blood glucose was estimated by following glucose oxidase method on UV- visible spectrophotometer Jenway 6305.The HbA_1c_ was analyzed by automatic analyzer (Backman Coulter, Italy). CRP level was determined by quantitative turbidimetric method using Spinreact test kit.

***Data Analysis: ***Data were statistically analyzed using Statistical Package for Social Sciences version 15.0 (SPSS Inc, Chicago, IL, USA). Independent samples were examined with student’s t test. P-values and 95% confidence intervals (CI) were also calculated. For all comparisons P-value of less than 0.05 was taken as statistically significant.

**Table-I:** Comparison between Control and Diabetic Subjects with respect to the, Fasting Blood Glucose, HbA1c, Platelet Indices and hs-CRP level.

*Parameters*	*Control* *(n = 55)*	*Diabetic Patients* *(n = 51)*	*P- Value*
FBG mg/dl	94.36 ± 1.82	164.4 ± 6.13	0.0001*
HbA1c %	4.79 ±0.052	6.8 ± 0.169	0.0001*
PLT 10^3^/mm^3^	244.82 ± 11.48	250.33 ± 11.53	0.737
PCT %	0.206 ± 0.0095	0.220 ± 0.0108	0.29
MPV µm^3^	8.535 ± 0.166	9.21 ± 0.14	0.0026*
PDW %	13.86 ± 0.297	14.71 ± 0.21	0.0269*
hs-CRP g/dl	2.103 ± 0.376	11.60 ± 3.13	0.0029*

Fasting blood glucose (FBG), Glycosylated hemoglobin (HbA_1_c), Platelet count (PLT), Plateletcrit (PCT), Mean platelet volume (MPV), Platelet distribution width (PDW), high sensitivity C - reactive protein (hs-CRP)

n = no of subjects, values are represented as mean ± SEM (Standard error of mean).

* P < 0.05 is considered to be statistically Significant.

## Results

**Fig.1:**
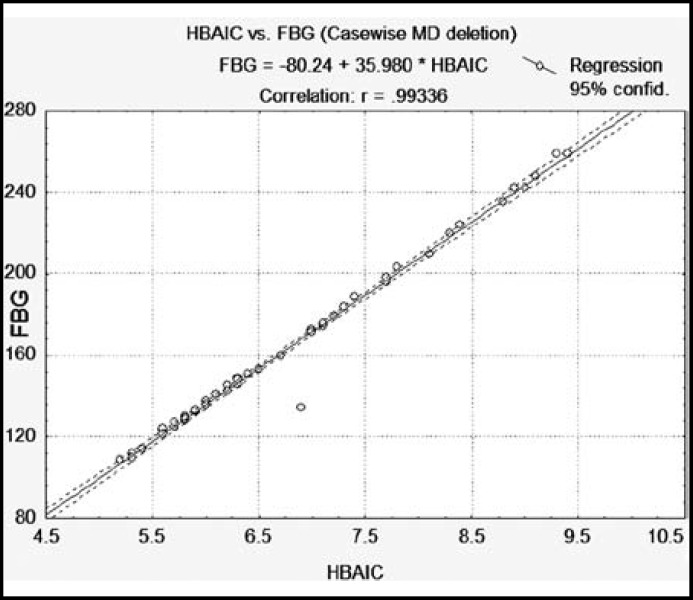
Scatterplot showing positive correlation between Fasting Blood Glucose (FBG) level with glycosylated hemoglobin (HbA1c) (r = 0.993, p = 0.0001).

**Fig.2:**
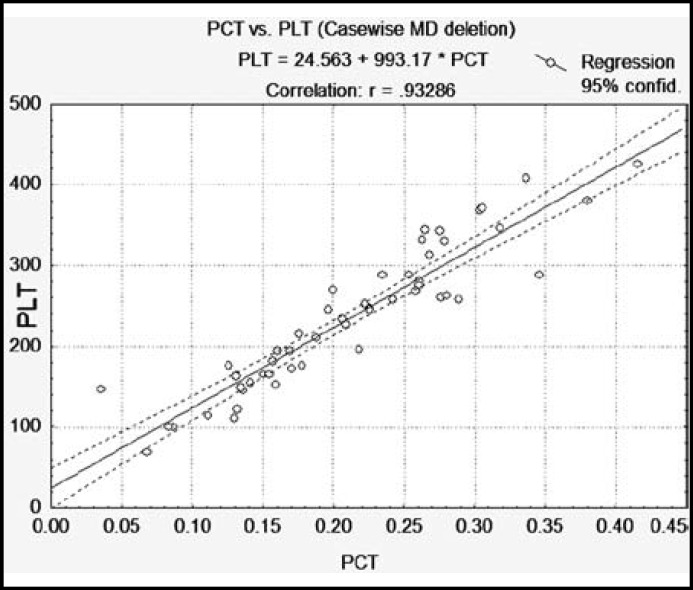
Scatterplot showing positive correlation between Platelet Count (PLT) with Plateletcrit (PCT). (r = 0.922, p = 0.00)

**Fig.3:**
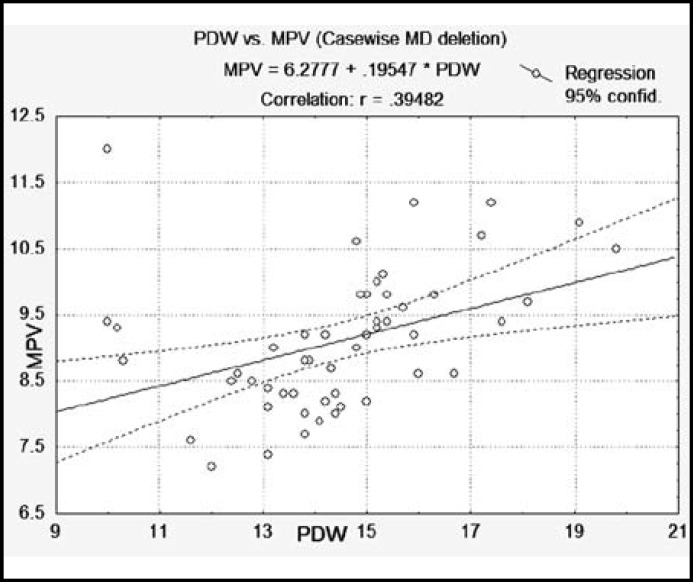
Scatterplot showing positive correlation between Mean Platelet volume (MPV) with Platelet distribution Width (PDW) (r = 0.332, p = 0.024).

 The study was based on 51 patients with diabetes and 55 healthy control subjects. The Mean age of control was 54 ± 7years and 52 ± 7years for patients. The results of biochemical constituents (FBG, HbA1c, and hs-CRP) and platelet indices were compared in patients and control subjects. Significant increase in FBG, HbA1c, MPV, PDW and CRP were found in diabetic patients as compared to healthy control subjects (Table-I). Coefficient of correlation was used to find the significant correlation among biochemical constituents as well as platelet indices in diabetic patients (Table-II). Positive correlations was found in glycemic control determinant (FBG with HbA1c) and platelet indices (PLT with PCT) and (MPV with PDW) (Fig. 1-3 scattered graph), and no significant correlation was found in hs-CRP level with glycemic control and platelet indices. The Variations of FBG, HbA1c, Platelet indices and hs-CRP levels in male and female control and Diabetic subjects are shown in (Table-III) indicating significant increase in FBG, HbA1c, MPV, PDW and CRP in diabetic male and female patients in comparison with control. Female patients had significantly high levels of MPV, PDW and hs-CRP in comparison with male diabetic patients.

**Table-II:** Correlations of Platelet Indices and Biochemical Parameters in Type-2 Diabetic Patients

		*PLT*	*PCT*	*MPV*	*PDW*	*FBG*	*HbA1c*	*CRP*
PLT: r	Pearson Correlation	1	.922(**)	-.126	-.086	.060	.067	-.017
p	Sig. (2-tailed)	.	.000	.417	.595	.701	.668	.913
N = 51								
PCT: r	Pearson Correlation	.922(**)	1	.247	.096	-.133	-.104	-.018
p	Sig. (2-tailed)	.000	.	.087	.529	.362	.477	.901
N = 51								
MPV: r	Pearson Correlation	-.126	.247	1	.332(*)	-.128	-.077	-.149
p	Sig. (2-tailed)	.417	.087	.	.024	.369	.593	.296
N = 51								
PDW: r	Pearson Correlation	-.086	.096	.332(*)	1	.027	-.012	.073
p	Sig. (2-tailed)	.595	.529	.024	.	.857	.938	.628
N = 51								
FBG: r	Pearson Correlation	.060	-.133	-.128	.027	1	.993(**)	-.089
p	Sig. (2-tailed)	.701	.362	.369	.857	.	.000	.535
N = 51								
HbA1c: r	Pearson Correlation	.067	-.104	-.077	-.012	.993(**)	1	-.095
p	Sig. (2-tailed)	.668	.477	.593	.938	.000	.	.509
N = 51								
hs-CRP: r	Pearson Correlation	-.017	-.018	-.149	.073	-.089	-.095	1
p	Sig. (2-tailed)	.913	.901	.296	.628	.535	.509	.
N = 51								

**Correlation is significant at the 0.01 level (2-tailed).

*Correlation is significant at the 0.05 level (2-tailed).

## Discussion

 The main objective was to determine the relationship between the glycemic control, platelet indices and CRP levels in patients of type II diabetes. Impaired fasting glucose is probably a frequent glycemic disorder in the general population, lead to hyperglycemia, whose injurious effects are categorized in to micro and macro vascular disorders. Inadequate glycemic control, protein glycation and oxidative stress cause endothelial injury and platelet activation with altered platelet morphology and function leading to chronic complications in diabetics.^8^ The prevalence of diabetic micro vascular complications is greater in people with poor control of glucose level, long term duration of diabetes, an elevated blood pressure and obesity. Glycosylated hemoglobin (HbAIc) has been validated as a reliable indicator to evaluate the metabolic state of diabetic patients.^9^

 Several studies indicate the platelet indices and CRP level as thrombogenic markers play a key role in the integrity of normal homeostasis.^10^ Our study reveals the significant increase in mean platelet volume (MPV) and platelet distribution width (PDW) with poor glycemic control and high CRP level (Table-I). Patients with larger platelets can easily be identified during routine hematological analysis whose measurements are based on impedance technology that measure platelet volume by deformation of electric field.^11^

 In the present study it was found that various parameters such as FBG, HbA1c, MPV, PDW and CRP were high in male and female patients as compared to control subjects (Table-III). Female diabetic patients revealed significantly high value of MPV, PDW and CRP in comparison with diabetic male patients suggesting them to develop thromboembolic complications in future. It’s also becoming clear that high levels of CRP may point to the presence of inflammation in blood vessels and be an early marker for heart disease. According to the new research finding the higher levels of hs-CRP associated with the susceptibility of developing Type 2 diabetes, especially in women.^12^ Several studies have revealed that CRP, ESR, PLT are potential mediators for atherogenesis and proved useful in finding of cardiovascular disease. The diagnostic use of these biomarkers will provide reliable, accurate, and cost-effective information to predict future thrombotic events especially in countries with poor socioeconomic status like Pakistan. Levels of hs-CRP in blood are now commonly used in medical practice to improve prediction of vascular risk.^13^

**Table-III:** Variations of FBG, HbA1c, CRP and Platelet Indices in Control and Diabetic Male and Female Subjects.

*Variables*	*Control (n=55)*		*Diabetic Patients (n=51)*	
	*Male (n= 22)*	*Female (n=33)*	*Male (n= 28)*	*Female (n=23)*
FBG mg/dl	90.34 ± 2.92	96.97 ± 2.24	*169 ± 8.91	*158 ± 8.21
HbA1c %	4.69 ± 0.08	4.86 ± 0.06	*6.91 ± 0.24	*6.65 ± 0.22
PLT 10^3^/mm^3^	224.55 ± 18.73	258.33 ± 14.25	252.36 ± 14.60	247.9 ± 18.70
PCT %	0.18 ± 0.01	0.22 ± 0.012	0.218 ± 0.01	0.22 ± 0.01
MPV µm^3^	8.38 ± 0.25	8.6 ± 0.21	*8.95 ± 0.16	*9.53 ± 0.23
PDW %	13.94 ± 0.37	13.81 ± 0.43	*14.41 ± 0.27	*15.15 ± 0.33
hs-CRP mg/dl	2.03 ± 0.61	2.14 ± 0.48	*8.92 ± 4.03	*14.86 ± 4.94

Fasting blood glucose (FBG), Glycosylated hemoglobin (HbA_1_c), Platelet count (PLT), Plateletcrit (PCT), Mean platelet volume (MPV), Platelet distribution width (PDW),

high sensitivity C - reactive protein (hs-CRP)

n = no: of subjects, values are represented as mean ± S.E.M (Standard error of mean).

* Statistically significant as compared to control.

## Conclusions

 Positive association between glycemic control determinant (FBG & HbAIc) and platelet indices (PLT-PCT) and (MPV-PDW) indicate the clinical usefulness of HbA_1c_ level and platelet indices as surrogate marker in early detection of diabetic complications. Although no significant correlation was found in CRP with glycemic control and platelet indices suggesting it as an independent important marker of inflammation that may contribute in finding micro and macrovascular complications in diabetics.

## Notes

***Source of funding:*** Jinnah University for Women Nazimabad, Karachi, Pakistan.
